# Maternal mortality due to abortion complications in forcibly displaced populations: A study protocol for a community-facility capture-recapture (CFCR) study

**DOI:** 10.1371/journal.pone.0315182

**Published:** 2025-02-28

**Authors:** Blake Erhardt-Ohren, Dipika Paul, Anik Mahmud, Anika Tarannum, Karen Weidert, Altaf Hossain, Sayed Rubayet, Ndola Prata

**Affiliations:** 1 Bixby Center for Population, Health, & Sustainability, School of Public Health, University of California, Berkeley, Berkeley, California, United States of America; 2 Ipas Bangladesh, Dhaka, Bangladesh; 3 Association for the Prevention of Septic Abortion, Bangladesh, Dhaka, Bangladesh; Jhpiego, UNITED STATES OF AMERICA

## Abstract

There is a paucity of research exploring abortion complication-related morbidity and mortality in humanitarian settings. The most recent data we have to understand the impact of global negligence on forcibly displaced persons’ reproductive health needs in humanitarian emergencies is from the 1999 United Nations Population Fund’s annual report, which estimated that 25-50% of maternal deaths in refugee settings were due to complications from unsafe abortion. This study will investigate maternal death surveillance and reporting (MDSR). The protocol will be implemented in a refugee setting: Forcibly Displaced Myanmar National (FDMN) camps in Cox’s Bazar, Bangladesh. We will review death records for the past twelve months to learn more about how deaths are reported and recorded in facilities and in the camp-in-charge (CiC) office. Following the record review, we will interview individuals who provide reproductive healthcare services to FDMN and participate in MDSR where FDMN reside. These interviews will provide context and depth to the maternal death record review. We will implement a novel community-facility capture-recapture (CFCR) methodology to estimate maternal mortality. This research will fill a gap in knowledge about menstrual regulation, safe abortion and post-abortion access and care, and the measurement of maternal death due to abortion-related complications. This study will provide insights into a new opportunity to potentially more accurately measure maternal mortality due to abortion complications in these settings. The evidence gathered in the course of this research may assist global health practitioners in targeting interventions to prevent unsafe abortion and increase access to safe services that are tailored to forcibly displaced populations. The University of California, Berkeley Center for Protection of Human Subjects (CPHS # 2016-04-8614) and the National Research Ethics Committee (NREC) of the Bangladesh Medical Research Council (BMRC) (Registration # 578 10 03 2024) approved this study protocol.

## Introduction

In 2019, approximately 218 million individuals of childbearing age had an unmet need for modern contraception, meaning they were sexually active, did not want a child now or ever, and were not using an effective method of contraception [1]. Even with contraceptive use, individuals may become pregnant due to user error or contraceptive failure. A recent comprehensive study, including data from 166 countries, estimated that from 2015-2019, there were 121 million unintended pregnancies annually, of which 48% were unintended [2]. The same study estimated that 61% of unintended pregnancies ended in abortion; this amounts to roughly 73 million induced abortions each year.

By the end of 2023, there were approximately 117.3 million forcibly displaced individuals around the world, including 37.6 million refugees, and almost one million (n =  961,801) Rohingya arriving in Bangladesh while fleeing religious persecution in Myanmar [3]. This marks an increase of Rohingya refugees of only one percent from the previous year but ~ 315% from the number in 2015, when the crisis began — the United Nations Refugee Agency expects this number to continue increasing. This is a global trend: the number of refugees globally has steadily increased since 2009 [3] and shows signs of acceleration due to increased internal conflict, intolerance, inequalities, and compounding factors such as famine, related starvation, and climate change [4]. Approximately one in four of these refugees is capable of pregnancy [3].

There is a paucity of research exploring abortion-related mortality in complex humanitarian emergencies. Instead, past investigations into maternal mortality due to abortion complications focus on general populations, individuals that receive treatment at a hospital before death, and/or sex worker populations [5–18]. A systematic review of these methodologies questioned their reliability [19]. The most recent data we have to understand the impact of global negligence on forcibly displaced persons’ reproductive health needs in humanitarian emergencies is from the United Nations Population Fund’s 1999 annual report, which estimated that 25-50% of maternal deaths in refugee settings were due to complications from unsafe abortion [20]. Meanwhile, researchers estimated that global maternal mortality due to unsafe abortion was 7.9% in 2003 to 2009, meaning the United Nations Population Fund’s estimate for refugees from 25 years ago represents an increased risk of about three to six times [21]. In 2007, the Working Group for Mortality Estimation in Emergencies called for new estimation methods. Still, there have been few responses [22–26], even fewer with a focus on maternal mortality [27,28], and none that report on abortion-related mortality.

The overall objective of this study is to fill a knowledge gap about the measurement of maternal death, especially due to abortion complications, within the Forcibly Displaced Myanmar National (FDMN) camps in Cox’s Bazar, Bangladesh. The specific objectives are to (1) investigate the process of recording maternal mortality by assessing maternal death records and interviewing maternal death surveillance and response (MDSR) focal points, (2) explore whether we can identify additional deaths to the health facility data by using community informants, and (3) determine what proportion of maternal deaths are due to abortion-related complications using a novel community-facility capture-recapture (CFCR) methodology.

## Materials and methods

### Study sites

There are 33 refugee camps in Cox’s Bazar, Bangladesh hosting 954,131 refugees as of 31 July 2024 [29]. We will purposively select five FDMN camps in Cox’s Bazar based on their population size and composition (i.e., demographic structure) and health infrastructure and composition (i.e., number and type of health facilities, health services offered). For the maternal death record review and MDSR focal points, we will purposively select at least ten health facilities (Primary Health Care Centers) with varying population bases. For the novel CFCR implementation, we will not focus on specific health facilities, but rather on the five camps as a whole.

### Research data collection

Two research assistants will be recruited in Cox’s Bazar to assist with data collection. These assistants will be adult women who can read maternal death records in Bangla, interview MDSR focal points in Bangla and Rohingya, and conduct verbal autopsies in Bangla and Rohingya. The assistants will receive two days of training on ethics and data collection at a hotel venue. The training will specifically cover the issue of abortion stigma and will include sensitivity training and guidance for how to collect data on taboo topics.

### Maternal death record review

A desk review of maternal death records at a selection of health facilities providing menstrual regulation (MR) – i.e., the induction of menstruation when it has been missing for twelve or fewer weeks without first checking for pregnancy — and post-abortion services to FDMN will examine how maternal cause of death is determined, where relevant information is stored, how it is classified, and who is involved in the process.

#### Sampling.

All records of deaths in the past twelve months at selected facilities will be considered eligible for review.

#### Data collection.

At the regular health facility meetings, researchers will coordinate with the health facility manager to pick a day a research assistant can review the last twelve months of maternal deaths at the facility and the camp-in-charge (CiC), who is the coordinator assigned to manage a specific FDMN camp. On the appointed day, the health facility manager will provide the research assistant with maternal death records for the last twelve months and provide any clarification on the records as needed. The health facility manager will be provided with contact information for the study group. Hence, the manager can reach out with any questions, comments, or concerns they may have in the future.

Data will be collected from the registers using an instrument. The following variables will be collected for all maternal deaths recorded in the preceding twelve months: date of death, age of decedent, location of death (facility, community, home), time of death (during pregnancy, within 42 days of delivery, between 43 days and twelve months after delivery), whether the individual was admitted to the hospital in the last twelve months, the probable cause of death, the role of the cause of death assignee, and any other notes related to the entry in the register. If these data are unavailable in the registers, they will not be recorded. Suppose a health facility uses a form other than the Ministry of Health and Family Welfare-issued death register. In that case, the data collection form may be adapted to the form used at the facility. However, no identifying information will be recorded. For example, if the cause of death is not listed in the death records, this will be a finding, and we will leave this portion of the form blank. The decedent’s name, address, and other specific identifying information will not be obtained. These data will be entered directly into Microsoft Excel version 16.80 on a locked laptop.

These review sessions will be conducted in a private room with a closed door in the health facility or the CiC office. This way, the records are not transported out of the facility, and others in the facility cannot see them as they are reviewed. The data will be stored on a password-protected laptop in an Excel file until it can be added to an online database that requires two-factor authentication.

#### Data analysis.

The maternal death record review will be used to better understand how deaths are currently classified at the health facilities and CiC in the FDMN camps, and which causes of death are most common. We will produce descriptive statistics using R version 2023.09.1 + 494.

### MDSR focal point interviews

We will interview MDSR focal points at facilities providing reproductive health services to FDMN about their knowledge of the MR program in Bangladesh, abortion law in Bangladesh and knowledge, attitudes, and practices around MR and abortion care service provision. Questions will probe perceived client-level, facility-level, and policy-level barriers and facilitators to MR and safe abortion service provision. A final set of questions will ask about how maternal deaths are recorded when they occur in the community and at a facility.

#### Study population and sampling.

A convenience sample will be taken to understand the processes for recording maternal deaths in the camps. Potential participants will be identified collaboratively by Ipas Bangladesh, the Association for Prevention of Septic Abortion, Bangladesh (BAPSA), the United Nations Refugee Agency, and/or members of other humanitarian organizations working in the Health Cluster of FDMN camps in Cox’s Bazar. Subjects will be identified to achieve diversity in the study population concerning roles in providing health care and participating in MDSR-related activities. Willingness to participate, being able to provide written consent to the study protocol, communicating in Bangla or Rohingya, and being of the age of majority in Bangladesh are overriding inclusion criteria. Being unwilling to participate and/or not ready or able to provide written consent are overriding exclusion criteria. Participants must work at an institution that provides reproductive health services to FDMN in Cox’s Bazar.

Participants will be approached via email and/or WhatsApp to their work accounts or at regularly scheduled health facility meetings to participate in the study. We will conclude interviews once the qualitative data reaches code and meaning saturation, which is expected around fifteen interviews but no more than 25.

#### Data collection.

The two research assistants will interview MDSR focal points over two weeks under the supervision of senior Ipas Bangladesh and Bixby Center staff. Each interview will be conducted in a private room within the health facility to ensure privacy and confidentiality. If the research assistants are unable to find a private, secluded space to conduct an interview, the interview will be postponed until confidentiality can be ensured.

Participants will be consented by a research assistant in the participants’ language of choice, Bangla or Rohingya. After they sign the consent form, the research assistant will ask if they may record the interview and explain what will happen to the recording. The research assistant will also explain that the participant may request to stop the interview anytime and for any reason. Interviews are expected to take between 30 and 45 minutes, at maximum. After the interview, the research assistant will ask if the participant has any questions and provide contact information for the study researchers. Hence, the participant can reach out with any questions, comments, or concerns they may have in the future.

Consent forms collect the participants’ names, signatures, and the date of the interview. After the consent form is signed, a research assistant requests permission to record the interview using an audio recording device. The research assistant does not ask the participants to identify themselves once the audio recording begins.

We do not anticipate that these data could be linked back to participants. We will assign each participant a number to link their interview to their consent form, which will be stored in a separate folder in an online database that requires two-factor authentication and is accessible only to the research team members. Only basic demographic information will be collected during interviews, and participants will be assigned a number instead of having their names documented. We will not collect information on the health facility name/organization that the individual works for. Once the interviews are completed, they will be transcribed and translated using one of Ipas Bangladesh and BAPSA’s trusted vendors. The audio recordings will be destroyed after transcription.

#### Data analysis.

Demographic data from healthcare worker interviews will be stored in Microsoft Excel version 16.80 and imported into R version 2023.09.1 + 494 to produce descriptive statistics about interview participants. The qualitative data will be transcribed and translated into Microsoft Word documents. These Word documents will be imported into MaxQDA version 24 for coding. We will create a deductive codebook based on the interview questions that we will then supplement with inductive codes that arise in our analysis.

### Verbal autopsy

The novel CFCR methodology will be utilized to estimate the number of maternal deaths in a specific period of time. Two data sources, one from health facilities and CiC offices one from community informants will be compared to establish the total number of deaths in each camp over three months. Verbal autopsies using an adapted questionnaire from the Bangladesh Maternal Mortality and Health Care Survey 2016 [30], will help to determine the cause of death in cases where it is unknown or considered suspicious.

#### Study population and sampling.

We will purposively select the FDMN camps based on their population size and composition (i.e., demographic structure) and health infrastructure and composition (i.e., number and type of health facilities and health services offered). Then, death records will be collected prospectively from all facilities and CiCs, in addition to community-based data collection.

All deaths to women of reproductive age, i.e., 15-49 years, in the three months will be included in the study. One next of kin for whom the decedent’s cause of death is unknown or suspicious, who is of the age of majority, and who consents to verbal autopsy will be invited to participate in the verbal autopsy. Research assistants will attempt to recruit an individual who was present at the time of death, preferably a cohabitant of the decedent, and female sex.

Willingness to participate, ability to consent to the study protocol, ability to communicate in Bangla or Rohingya, and being of the age of majority in Bangladesh are overriding inclusion criteria. Being unwilling to participate and/or not ready or able to consent are overriding exclusion criteria. Participants must be related to a decedent whose death is included in the study.

#### Data collection.

For three months, a research assistant will collect maternal death data from each health facility and CiC in selected FDMN camps bi-weekly. Community leaders will be selected based on their prominence within the community and residence within a specific camp.

Selected community leaders will meet bi-weekly with the research assistant to report on deaths that they are aware of among individuals during pregnancy, delivery, or in the 42 days following delivery.

The research assistant will then compile the full list of deaths. If any deaths appear to be pregnancy-related or maternal deaths for which there is no assigned cause of death in the health facility or CiC registers, or if the cause of death appears to be abortion-related, the research assistant will attempt three times to contact a next of kin at the family residence of the decedent in the following week. The research assistant will contact the available next of kin; if multiple individuals are present, one female adult will be randomly selected to conduct a verbal autopsy.

The meetings between the research assistant and community informants will be conducted in a private room with a closed door in the health facility or the CiC office to maintain the confidentiality of decedents. This way, the records are not transported out of the facility, and others cannot see them when they are reviewed. Verbal autopsies will be conducted in private and secluded spaces. If research assistants cannot find a private, secluded space to conduct an interview, the interview will be postponed until the time at which confidentiality may be ensured. These data will be entered directly into Microsoft Excel version 16.80 on a locked laptop.

Participants in the community leader and verbal autopsy groups will be compensated for their time, commensurate with appropriate compensation in this location.

#### Data analysis.

**Fig 1:**
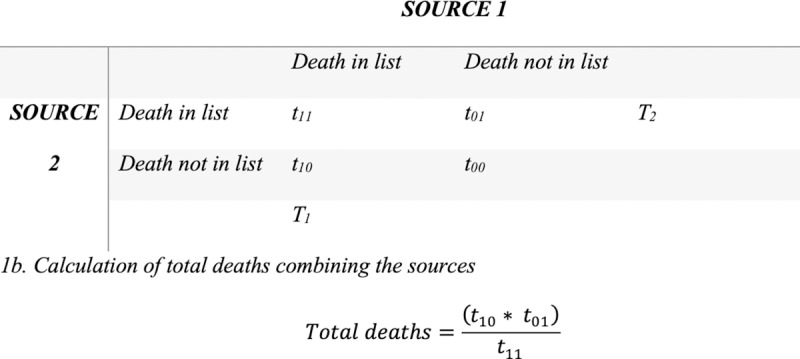
Quantifying the total number of maternal deaths and/or deaths due to abortion-related complications using the Lincoln-Petersen Estimator. (A) *Variable names in each source. (B) Calculation of total deaths combining the sources.*

Every three weeks, a researcher will enter the data into R version 2023.09.1 + 494 to produce descriptive statistics on the deaths from the health facilities/CiC and community leaders. The researcher will calculate the number of deaths collected from the facilities/CiC and community aspect and compare the characteristics of each death to confirm if they are fully matched, partially matched, or unmatched in the two data sources. We will base matching on the following characteristics of the individual: name, age, address, and date of death. We will consider deaths to be matched fully if three or all characteristics are the same in both sources. We will consider deaths to be partially matched if only two characteristics match. If only one characteristic matches, we will consider deaths unmatched. We will estimate the “true” number of maternal deaths and the proportion of those deaths that are due to MR/PAC complications using the classic Lincoln-Petersen estimator for total population [31] (see Fig 1). This value will be compared to the number of suspected pregnancy-related and maternal deaths in health facility registers, which is a common way to estimate maternal mortality in low- and middle-income countries. This will help us to understand whether the new methodology allows us to capture any additional pregnancy-related or maternal deaths than there are in facility- or hospital-based records.

### Data storage

For all components, electronic records will be secured on password-protected computers until they can be uploaded to CalShare, a University of California, Berkeley-operated Microsoft SharePoint platform. The University approves the system for storing sensitive data, including “critical data” that may contain personally identifiable human subject data. It includes data encryption whenever data are in transit. The system requires dual-factor authentication. We do not anticipate keeping any physical records. Study researchers will have access to study records. No information will be shared with individuals outside the study research team until it is properly de-identified. Anonymized study data will be archived. Data that may be linked back to study participants will be destroyed within six months of data collection. Audio files will be initially stored on the research assistant’s recording device. Still, they will be downloaded to their password-protected laptop and encrypted at the end of each day of interactions. When the schedule permits, transfers will take place immediately following an interview.

Once saved to the password-protected laptop, audio files will be deleted from the recording device. Transfer of encrypted audio files from the password-protected laptop to CalShare will occur. At this point, encrypted audio files will be deleted from the researchers’ password-protected laptop. The encrypted files on the password-protected server will be uploaded to a secure website of a professional transcription service. This will take place within one week of the interviews being conducted. The transcription service will name the word files using the anonymous interview identifier numbers. Once the transcription service completes transcripts — usually within five workdays — they will be reviewed for accuracy by bilingual research team members. At this point, the associated audio file will be deleted. Transcripts received from the professional transcription service will be encrypted and stored on the CalShare.

### Ethics approval

This study protocol was approved by the University of California, Berkeley Center for Protection of Human Subjects (CPHS # 2016-04-8614) and the National Research Ethics Committee (NREC) of the Bangladesh Medical Research Council (BMRC) (Registration # 578 10 03 2024).

### Timeline

We expect to begin this project in December 2024 with an end date no later than August 2025.

### Limitations

This study will have some limitations. While the standard for reporting maternal mortality is the maternal mortality ratio, we plan to investigate abortion in the structure of causes of maternal death. Therefore, we will be reporting maternal mortality without a denominator (live births). This is because of our small sample size and our desire to test the feasibility of the CFCR methodology before a second phase where we plan to expand the project to multiple sites with larger populations. Secondly, abortion is a sensitive topic. While we plan to provide sensitivity training to research assistants, there is likely to be under-reporting of abortion-related deaths. For this reason, we will report the proportion of deaths that we believe are abortion-related with a confidence interval, instead of providing only a point estimate. The Lincoln-Petersen Estimator for capture-recapture estimation relies on three assumptions: (1) independence between sources, (2) a closed population, and (3) independence between individuals.[31] While we make these three assumptions in this initial test, we hope to further explore assumptions in the future.

## Discussion

Many methodologies have been developed to measure maternal mortality due to various causes. These methodologies may be grouped in several different ways by the opportunity in which the method is deployed (routine, special opportunity, composite, analytical), method type (longitudinal and continuous capture of deaths, cross-sectional capture, mixed approach, no new capture of deaths) [32] but are most commonly grouped by approach: either direct methods, which require the use of vital registration or hospital data or indirect methods which may use vital registration or hospital data, but rely on other techniques to elicit information about maternal deaths. Examples of direct methods include the capture-recapture method, direct sisterhood method, maternal death records review, and vital/civil registration review. Examples of indirect methods include community health worker surveillance, community knowledge method, indirect sisterhood method, MADE IN/MADE FOR method, motherhood method, neighborhood method, reproductive age mortality (RAMOS) method.

Each of these methods carries various biases that affect their accuracy. Registration-based methods may not find data representative of the population at large due to inaccurate reporting of deaths and misclassification of cause of death [33]. Methods that rely on sibling, including sister (sisterhood method), or neighbor reports of maternal death make key three assumptions that may be violated: that decedents have a living sibling, that decedents told their sibling about an abortion before their death, and that the sibling was present at the death [34]. Not all of these methods lead to estimates of deaths due to abortion complications. When they do, however, they have the same potential for biased results [19].

Maternal mortality due to abortion complications is a pressing issue around the world that is amplified in humanitarian emergencies. The number of forcibly displaced persons is accelerating each year and shows no sign of slowing down. This means the number of forcibly displaced individuals of childbearing age is increasing each year, meaning more unintended pregnancies and more unsafe abortions unless issues of access to services are addressed. We cannot rely on an estimate of maternal death from unsafe abortion complications in forcibly displaced settings that was generated almost 25 years ago. Nor can we rely on global estimates and assume that the proportion of maternal mortality due to unsafe abortion is the same in stable and humanitarian emergency settings. We cannot rely on extrapolations about access to health services from general populations to humanitarian emergency contexts. This lack of research and evidence contributes to the ongoing problem.

This is a human rights issue: access to abortion services empowers individuals to decide if, when, and how many children to have. In 1994, at the International Conference on Population and Development (ICPD), leaders around the world defined reproductive rights and what they entail:

“… basic right of all couples and individuals to decide freely and responsibly the number, spacing and timing of their children and to have the information and means to do so, and the right to attain the highest standard of sexual and reproductive health. It also includes the right of all to make decisions concerning reproduction free of discrimination, coercion and violence as expressed in human rights documents.” [35]

We expect there to be two main contributions that the results of this study will make to health development in Bangladesh and refugee camps around the world:

1. The maternal death record reviews and interviews with MDSR focal points will shed light on record-keeping in FDMN camps, allowing for improved monitoring and evaluation of deaths in the future, so programs may be better tailored to the reduction of unnecessary maternal death.
2. Reliable tools are needed to estimate maternal mortality in humanitarian settings. We expect that this research will result in the production of a guidebook for individuals to implement similar methodologies to this study to monitor maternal mortality in FDMN camps in Cox’s Bazar, Bangladesh, at a reduced cost compared to other methodologies.

This proposed research will provide a new estimate of maternal death from abortion complications in FDMN camps in Cox’s Bazar, Bangladesh, providing a benchmark against which reproductive health programs can measure progress. It will also result in the creation of a tool to assist with monitoring and evaluating reproductive health programs targeting a reduction in maternal mortality due to unsafe abortion. This proposed research is significant because it uplifts individuals whose healthcare needs have historically been marginalized, provides evidence for safe MR and abortion service advocacy, and will contribute to future endeavors to implement successful maternal mortality surveillance activities and measurement camps hosting forcibly displaced persons.

## Supporting information

S1 File Maternal death record review instrument. (DOCX)

S2 File Interview guide questions. (DOCX)

S3 File Verbal autopsy tool. (DOCX)
