# Supplementary material for: Maternal mortality due to abortion complications in forcibly displaced populations: A study protocol for a community-facility capture-recapture (CFCR) study
Source: PLoS One. 2025 Feb 28;20(2):e0315182. doi: 10.1371/journal.pone.0315182 (PMC11870353; doi:10.1371/journal.pone.0315182)
Supplement: S1 File — (DOCX) [file pone.0315182.s001.docx]

**S1. Maternal death record review instrument**

Health facility/camp name:

Date of review:

Dates under review:

| **#** | **Date of death** | **Location of death** | | | **Time of death** | | | **Admitted to hospital in last 12 months** | **Probable cause of death** | **Role of person assigning COD** | **Notes** |
| --- | --- | --- | --- | --- | --- | --- | --- | --- | --- | --- | --- |
|  |  | **Facility** | **Comm.** | **Home** | **During pregnancy** | **Within 42 days of delivery** | **Between 43 days and 12 months after delivery** |  |  |  |  |
| 1 |  |  |  |  |  |  |  |  |  |  |  |
| 2 |  |  |  |  |  |  |  |  |  |  |  |
| 3 |  |  |  |  |  |  |  |  |  |  |  |
| 4 |  |  |  |  |  |  |  |  |  |  |  |
| 5 |  |  |  |  |  |  |  |  |  |  |  |
| 6 |  |  |  |  |  |  |  |  |  |  |  |
| 7 |  |  |  |  |  |  |  |  |  |  |  |
| 8 |  |  |  |  |  |  |  |  |  |  |  |
| 9 |  |  |  |  |  |  |  |  |  |  |  |
| 10 |  |  |  |  |  |  |  |  |  |  |  |
| 11 |  |  |  |  |  |  |  |  |  |  |  |
