# Supplementary material for: Maternal mortality due to abortion complications in forcibly displaced populations: A study protocol for a community-facility capture-recapture (CFCR) study
Source: PLoS One. 2025 Feb 28;20(2):e0315182. doi: 10.1371/journal.pone.0315182 (PMC11870353; doi:10.1371/journal.pone.0315182)
Supplement: S2 File — (DOCX) [file pone.0315182.s002.docx]

**S2. Interview guide questions**

**Demographics**

- Do you identify as male, female, or another gender?
- How old are you?
- What is your marital status?
- What type of healthcare provider or administrator are you?
- How long have you been working at [facility]?

**Warm-up**

1. How would you describe your current professional role?
2. What does a typical day look like for you at work?

**Reproductive health policies/laws**

1. Can you tell me about current laws related to reproductive health in Bangladesh?
   1. Current laws related to contraception/family planning
   2. Current laws related to safe MR and post-abortion care
2. How do current policies affect the healthcare needs of individuals in Bangladesh?
3. Under the law, who is able to access MR/PAC services?
4. Can you tell me about how these laws and policies affect forcibly displaced Myanmar nationals differently than other women?

**Current practice with unintended pregnancy**

1. What typically happens in Bangladesh when a woman finds out that she has an unintended pregnancy?
   1. How is this different for someone who is a forcibly displaced Myanmar national?
2. Where do women go for care when they have unintended pregnancies?
3. How is this different for someone who is a forcibly displaced Myanmar national?
4. How are women with unintended pregnancies counseled where you work?
5. What questions do healthcare workers ask the woman?
6. How do you feel about your workplace's role in counseling to women with an unintended pregnancy?
7. How is counseling different for forcibly displaced Myanmar nationals?
8. How are women provided with induced MR services where you work?
9. When are women provided with induced MR services?
   1. Gestational age
   2. Reason(s) given for termination
10. How do you feel about your workplace’s role in providing induced MRs to women?
11. What are some of the challenges and opportunities in the provision of induced MR?
12. How are services different for forcibly displaced Myanmar nationals?
13. How are women provided with post abortion care services where you work?
14. When are women provided with post abortion care services?
15. How do you feel about your workplace’s role in providing post abortion care services to women?
16. What are some of the challenges and opportunities in the provision of induced MR?
17. How are services different for forcibly displaced Myanmar nationals?

**Maternal death records**

1. How are maternal deaths in the community reported?
   1. Who is responsible for reporting deaths in the community?
   2. When there is a death in the community, is it reported immediately to the health facility or is there a delay?
   3. Who, at the facility, receives reports of deaths in the community?
   4. When there is a death in the community, is the cause of death assigned?
   5. Who determines the cause of death?
   6. Does someone at the facility assign whether the cause of death is an avoidable death?
   7. Is anything done to verify the cause of death?
2. How are maternal deaths in the facility documented?
   1. When there is a death in the facility, who is responsible for recording the death?
   2. When there is a death in the facility, is the cause of death assigned?
   3. Who determines the cause of death?
   4. Does someone at the facility assign whether the cause of death is an avoidable death?
   5. Is anything done to verify the cause of death?
3. What kind of training or guidance, if any, have you been provided with on how to report maternal deaths?
   1. Are there any special reporting procedures if the death is due to an MR/PAC or a suspected MR/PAC?
4. What makes it easier or harder to report maternal death accurately?
   1. In the community?
   2. At a facility?
5. When was the last time a maternal death occurred?
   1. What was the process of reporting, documenting, and assessing the death?

**Wrap-up**

1. Is there anything else that you think I should know before we end the interview?
