# Supplementary material for: Maternal mortality due to abortion complications in forcibly displaced populations: A study protocol for a community-facility capture-recapture (CFCR) study
Source: PLoS One. 2025 Feb 28;20(2):e0315182. doi: 10.1371/journal.pone.0315182 (PMC11870353; doi:10.1371/journal.pone.0315182)
Supplement: S3 File — (DOCX) [file pone.0315182.s003.docx]

**S3. Verbal autopsy tool**

We will utilize the verbal autopsy instrument from the *Bangladesh Maternal Mortality*

*and Health Care Survey 2016*, copied below.

SECTION 5

DETERMINING ELIGIBILITY FOR INTERVIEW MODULES 1-3

| ***NO.*** | ***QUESTIONS AND FILTERS*** | ***CODING CATEGORIES*** | ***SKIP*** |
| --- | --- | --- | --- |
| *5001* | *Was the woman pregnant at the time of death?* | *YES* *1*  *NO* *2*  *PROBABLY YES* *3*  *DON’T KNOW* *8* | *5004*  *5004* |
| *5002* | *How many months was she pregnant at the time of death?* | *MONTH* *\|* *\|* *\|*  *DON’T KNOW* *98* |  |
| *5003* | *Did the woman die before labour pain began or did she die after labour* | *MOTHER DIED BEFORE LABOUR BEGAN.. 1* | *6101* |
|  | *pain began?* | *MOTHER DIED AFTER LABOUR BEGAN* |  |
|  |  | *BUT BEFORE BIRTH OF CHILD* *2* | *7101* |
| *5004* | *Was*  *(NAME) ever pregnant while still alive?* | *YES* *1*  *NO* *2* | *8001* |
| *5005* | *What was the outcome of her last pregnancy?* | *LIVE BIRTH* *1* |  |
|  |  | *STILL BIRTH* *2* |  |
|  |  | *ABORTION/MISCARRIAGE/MR* *3* | *5006* |
|  |  | *DO NOT KNOW/UNSURE…* *8* | *5006* |
| *5005a* | *Was the baby born more than one month early?* | *YES* *1*  *NO* *2*  *DON’T KNOW* *8* |  |
| *5006a* | *What was the month and year of outcome of her last pregnancy?* | *MONTH* *\|* *\|* *\|*  *DON’T KNOW MONTH* *98*  *YEAR \|* *\|* *\|* *\|* *\|*  *DON’T KNOW YEAR* *9998* |  |
| *5006* | *How long after her delivery/last birth/still birth/abortion/miscarriage/MR did she die? If time is less than 1 day then write 00 days. If time is less than 2 months then write in completed days, if between 2 and 23 months then write in completed months, and if the time between pregnancy outcome and death is 24 months or more then write in completed years.* | *DAYS........................... 1 \|* *\|* *\|*  *MONTHS ..................... 2 \|* *\|* *\|*  *YEARS ........................ 3 \|* *\|* *\|*  *DON'T KNOW/UNSURE* *998* |  |
| *5007* | **Interviewer: Check answer to Q5006** | *Less than 12 months* *1*  *12 months (1 year) or more* *2* | *8001* |
| ***5008*** | **Interviewer: Check answer to Q5005 and circle the appropriate** | ***Q5005 IS CODED EITHER 1 OR 2*** ***1*** | ***7101*** |
|  | **code:** | ***Q5005 IS CODED EITHER 3 OR 8*** ***2*** | ***6102*** |

SECTION 6

MODULE 1: FOR DEATHS DURING PREGNANCY PRIOR TO ONSET OF LABOUR OR WITHIN 1 YEAR OF ABORTION/MISCARRIAGE/MR

| ***NO.*** | ***QUESTIONS AND FILTERS*** | ***CODING CATEGORIES*** | ***SKIP*** |
| --- | --- | --- | --- |
| *6101* | *Did*  *(NAME) ever see anyone for a medical checkup during that pregnancy?* | *YES* *1*  *NO* *2*  *DON’T KNOW* *8* | *6103*  *6201*  *6201* |
| *6102* | *Did*  *(NAME) ever see anyone for a medical checkup during the last pregnancy before she died?* | *YES* *1*  *NO* *2*  *DON’T KNOW* *8* | *6201*  *6201* |
| *6103* | *From whom did she receive the medical checkup when she was pregnant?*  *IF YES: Whom did she see?*  *Anyone else?*  *PROBE TO IDENTIFY EACH TYPE OF PERSON AND RECORD ALL MENTIONED.*  *IF CODE ‘D’ CIRCLED (WRITE NAME OF CSBA)* | *HEALTH PERSONNEL* |  |
|  |  | *QUAL. DOCTOR* *A* |  |
|  |  | *NURSE/MIDWIFE/PARAMEDIC…* *B* |  |
|  |  | *FAMILY WELFARE VISITOR…* *C* |  |
|  |  | *COMMUNITY SKILLED* |  |
|  |  | *BIRTH ATTENDANT…* *D* |  |
|  |  | *MA/SACMO…* *E* |  |
|  |  | *COMMUNITY HEALTH* |  |
|  |  | *CARE PROVIDER…* *F* |  |
|  |  | *HEALTH ASST…* *G* |  |
|  |  | *FAMILY WELFARE* |  |
|  |  | *ASSISTANT…* *H* |  |
|  |  | *NGO WORKER.* *I* |  |
|  |  | *OTHER PERSON* |  |
|  |  | *TRAINED TBA…* *J* |  |
|  |  | *UNTRAINED TBA…* *K* |  |
|  |  | *UNQUALIFIED DOCTOR…* *L* |  |
|  |  | *OTHER*  *X* |  |
|  |  | *(SPECIFY)* |  |
| *6104* | *Did she first seek a medical check-up during her last pregnancy* | *BECAUSE OF PROBLEM ONLY* *1* |  |
|  | *because she had a problem or just for a checkup?* | *FOR CHECK UP ONLY* *2* | *6106* |
|  |  | *FOR BOTH* *3* |  |
|  |  | *DON’T KNOW* *8* | *6106* |
| *6105* | *For what problem did she first seek medical check-up during her last pregnancy?*  *Verbatim*  *(1)*  *(2)* | *\|* *\|* *\| \|* *\|* *\|*  *DON'T KNOW/UNSURE* *98* |  |
| *6106* | *How many months pregnant was she at the time of her first medical check-up during her last pregnancy?* | *MONTHS* *\|* *\|* *\| DON'T KNOW/UNSURE* *98* |  |
| *6107* | *How many times did she get medical check-up during her last pregnancy?* | *NUMBER OF TIMES* *\|* *\|* *\| DON'T KNOW/UNSURE* *98* |  |
| *6201* | *Did she have swelling around her ankles during her pregnancy?* | *YES* *1*  *NO* *2*  *DON’T KNOW* *8* |  |
| *6202* | *Did she have puffiness of the face during her pregnancy?* | *YES* *1*  *NO* *2*  *DON'T KNOW/UNSURE* *8* |  |
| *6203* | *Did she complain of blurred vision during her pregnancy?* | *YES* *1* |  |
|  |  | *NO* *2* | *6204* |
|  |  | *DON’T KNOW* *8* | *6204* |
| *6203a* | *During the last 3 months of pregnancy did she suffer from blurred vision?* | *YES* *1*  *NO* *2*  *DON’T KNOW* *8* |  |

| **NO.** | **QUESTIONS AND FILTERS** | **CODING CATEGORIES** | **SKIP** |
| --- | --- | --- | --- |
| 6204 | Did she have her blood pressure measured during her pregnancy? | YES 1 |  |
|  |  | NO 2 | 6206 |
|  |  | DON’T KNOW 8 | 6206 |
| 6205 | Do you know whether her blood pressure was normal or high or low? | NORMAL 1  HIGH 2  LOW 3  DON’T KNOW 8 |  |
| 6206 | Did she have any loss of consciousness during that pregnancy? | YES 1 |  |
|  |  | NO 2 | 6207 |
|  |  | DON’T KNOW 8 | 6207 |
| 6206a | For how many days did she have loss of consciousness? | \| \| \|  days  DON'T KNOW/UNSURE 9998 |  |
| 6206b | Did the unconsciousness continue until death? | YES 1  NO 2  DON’T KNOW 8 |  |
| 6207 | Did she have fits (convulsions) during that pregnancy? | YES 1 |  |
|  |  | NO 2 | 6209 |
|  |  | DON’T KNOW 8 | 6209 |
| 6208 | How many days/months before her death did the fits start?  *(Write in months and days. If less than 1 month, then write 00 for months and only write in days)* | START \| \| \| \| \| \|  months days  DON'T KNOW/UNSURE 9998 |  |
| 6209 | Did she have headache during that pregnancy? | YES 1 |  |
|  |  | NO 2 | 6301 |
|  |  | DON'T KNOW/UNSURE 8 | 6301 |
| 6210 | Was the headache continuous or on and off? | CONTINUOUS 1  ON AND OFF 2  DON'T KNOW/UNSURE 8 |  |
| 6211 | How was the headache; severe, moderate, mild, or sometimes mild and sometimes severe? | SEVERE 1  MODERATE 2 |  |
|  |  | MILD 3 |  |
|  |  | SOMETIMES MILD AND SOMETIMES SEVERE  . 4 |  |
|  |  | DON'T KNOW/UNSURE 8 |  |
| 6301 | Did (NAME) have fever during that pregnancy or before her | YES 1 |  |
|  | death? | NO 2 | 6306 |
|  |  | DON’T KNOW 8 | 6306 |
| 6302 | How many days/months before her death did the fever start and end?  *(Write in months and days. If less than 1 month, then write 00 for months and only write in days)* | START \| \| \| \| \| \|  months days  END \| \| \| \| \| \|  months days |  |
|  |  | DIED WITH FEVER 9995 |  |
|  |  | DON'T KNOW/UNSURE 9998 |  |
| 6303 | How was the fever like? high or mild? | HIGH 1  MILD 2  DON'T KNOW/UNSURE 8 |  |

| **NO.** | **QUESTIONS AND FILTERS** | **CODING CATEGORIES** | | | **SKIP** |
| --- | --- | --- | --- | --- | --- |
| 6304 | Was the fever continuous or on and off? | CONTINUOUS 1  AFTER EVERY 1 - 2 DAYS 2  AT NIGHT ONLY 3  OTHER(specify)  . 7  DON'T KNOW/UNSURE 8 | | |  |
| 6305 | Did the fever come with severe chills? | YES 1  NO 2  DON'T KNOW/UNSURE 8 | | |  |
| 6306 | Did the colour of her eye change to yellow (jaundice) during that pregnancy? | YES 1  NO 2  DON'T KNOW/UNSURE 8 | | |  |
| 6307 | Did she have itching of skin at any time during that pregnancy? | YES 1  NO 2  DON'T KNOW/UNSURE 8 | | |  |
| 6308 | Did her eyes, face or palms look pale (anaemic) during that pregnancy? | PALE EYES PALE FACE PALE PALM | | **YES NO DK**  1 2 8  1 2 8  1 2 8 |  |
| 6309 | Did she have a cough during that pregnancy? | YES 1 | | |  |
|  |  | NO 2 | | | 6313 |
|  |  | DON’T KNOW 8 | | | 6313 |
| 6310 | How many days or months before her death did the cough start?  *(Write in months and days. If less than 1 month, then write 00 for months and only write in days* | START \| \| \|  months  DON'T KNOW/UNSURE | | \| \| \|  days  9998 |  |
| 6311 | Did the cough produce sputum? | YES 1  NO 2  DON'T KNOW/UNSURE 8 | | |  |
| 6312 | Did she cough blood? | YES 1  NO 2  DON'T KNOW/UNSURE 8 | | |  |
| 6313 | Did she have difficulty in breathing during that pregnancy? | YES 1 | | |  |
|  |  | NO 2 | | | 6319 |
|  |  | DON’T KNOW 8 | | | 6319 |
| 6314 | Was the difficulty in breathing continuous or on and off? | CONTINUOUS 1  ON AND OFF 2  DON'T KNOW/UNSURE 8 | | |  |
| 6315 | How many days/months before her death did the difficulty in breathing start and end? | START | \| \| \|  months | \| \| \|  days |  |
|  | *(Write in months and days. If less than 1 month, then write 00 for months and only write in days* | END | \| \| \|  months | \| \| \|  days |  |
|  |  | DID NOT IMPROVE/Continued until death 9995 | | |  |
|  |  | DON'T KNOW/UNSURE 9998 | | |  |
| 6316 | Was she breathless even on light work?  *(Except what is normally seen in late pregnancy, if applicable)* | YES 1  NO 2  DON'T KNOW/UNSURE 8 | | |  |
| 6317 | Was she breathless on lying on her back?  *(Except what is normally seen in late pregnancy, if applicable)* | YES 1  NO 2  DON'T KNOW/UNSURE 8 | | |  |

| **NO.** | **QUESTIONS AND FILTERS** | **CODING CATEGORIES** | **SKIP** |
| --- | --- | --- | --- |
| 6318 | Was there pain in the chest with breathing? | YES 1  NO 2  DON’T KNOW 8 |  |
| 6319 | Did she have palpitations during that pregnancy? | YES 1  NO 2  DON'T KNOW/UNSURE 8 |  |
| 6320 | Did she have chest pain during that pregnancy? | YES 1  NO 2  DON’T KNOW 8 | 6326  6326 |
| 6321 | Was the pain mild, moderate or severe? | SEVERE 1  MODERATE 2  MILD 3  DON'T KNOW/UNSURE 8 |  |
| 6322 | Did the pain start suddenly or gradually? | SUDDENLY 1  GRADUALLY 2  DON'T KNOW/UNSURE 8 |  |
| 6323 | Was the pain continuous or on and off? | CONTINUOUS 1  ON AND OFF 2  DON'T KNOW/UNSURE 8 |  |
| 6324 | How many days/months before her death did the pain start and end?  *(Write in months and days. If less than 1 month, then write 00 for months and only write in days)* | START \| \| \| \| \| \|  months days  END \| \| \| \| \| \|  months days  DID NOT IMPROVE/ Continued until death 9995 DON'T KNOW/UNSURE 9998 |  |
| 6325 | When she had the chest pain, did she also have pain elsewhere in her body? If, yes, where else did she have pain at the same time? | SHOULDER A  NECK B  ARMS C  NO PAIN ANYWHERE D  OTHER X |  |
| 6326 | Did she have abdominal pain during that pregnancy before her death? | YES 1  NO 2  DON’T KNOW 8 | 6329  6329 |
| 6327 | How many days/months before her death did the abdominal pain start and end?  *(Write in months and days. If less than 1 month, then write 00 for months and only write in days)* | START \| \| \| \| \| \|  months days  END \| \| \| \| \| \|  months days  DID NOT IMPROVE/ Continued until death9995 DON'T KNOW/UNSURE 9998 |  |
| 6328 | Was the pain mild, moderate or severe? | SEVERE 1  MODERATE 2  MILD 3  SOMETHIMES MILD/SOMETIMES MORE 4  DON'T KNOW/UNSURE 8 |  |
| 6329 | Was there any change in the color of her urine during that pregnancy before death? | YES 1  NO 2  DON’T KNOW 8 | 6331  6331 |

| ***NO.*** | ***QUESTIONS AND FILTERS*** | ***CODING CATEGORIES*** | | | | | ***SKIP*** |
| --- | --- | --- | --- | --- | --- | --- | --- |
| *6330* | *What color did the urine become?* | *LIGHT YELLOW* *1*  *DARK YELLOW* *2*  *CHUNER PANI (CLOUDY)* *3*  *BHATER MAAR (THICK-WHITE)* *4*  *BLOOD STAINED/RED* *5*  *OTHER (specify* *) . 7*  *DON'T KNOW/UNSURE* *8* | | | | |  |
| *6331* | *Was there any change in her daily frequency of urine during that pregnancy before her death?* | *YES* *1*  *NO* *2*  *DON’T KNOW* *8* | | | | | *6401*  *6401* |
| *6332* | *Compared to before, how many times was she passing urine in a day - more than before, less than before, or no urine at all?* | *MORE THAN BEFORE* *1*  *LESS THAN BEFORE* *2*  *NO URINE AT ALL* *3*  *DON'T KNOW/UNSURE* *8* | | | | |  |
| *6333* | *Since how many days/months before her death did she start to pass urine*  *(ANSWER TO Q6332)?*  *(Write in months and days. If less than 1 month, then write 00 for months and only write in days)* | *START \|* *\|* *\| \|* *\|* *\|*  *months days*  *DON'T KNOW/UNSURE* *9998* | | | | |  |
| *6401* | *During her last illness, did she have leaking membrane or did her water break?* | *YES* *1*  *NO* *2*  *DON’T KNOW* *8* | | | | | *6404*  *6404* |
| *6402* | *How many days/months before her death did she have leaking membrane or her water break?*  *(If less than 1 day then write in hours, if less than 30 days write in days and if more, then in completed months)* | *HOURS ....................... 1 \|* *\|* *\|*  *DAYS........................... 2 \|* *\|* *\|*  *MONTHS..................... 3 \|* *\|* *\|*  *DON'T KNOW/UNSURE* *9998* | | | | |  |
| *6403* | *Was she in pain when she had leaking membrane or when her water broke?* | *YES* *1*  *NO* *2*  *DON’T KNOW* *8* | | | | |  |
| *6404* | *Did she have other episodes of leaking membrane during her last pregnancy?* | *YES* *1*  *NO* *2*  *DON’T KNOW* *8* | | | | | *6501*  *6501* |
| *6405* | *Were these episodes of leaking membrane during her last pregnancy painful?* | *YES* *1*  *NO* *2*  *DON’T KNOW* *8* | | | | |  |
| *6501* | *During her last illness, was she bleeding from the vagina?* | *YES* *1*  *NO* *2*  *DON’T KNOW* *8* | | | | | *6502*  *6502* |
| *6501a* | *Was there vaginal bleeding during the first 6 months of pregnancy?* | *YES* *1*  *NO* *2*  *DON’T KNOW* *8* | | | | |  |
| *6501b* | *Was there vaginal bleeding during the last 3 months of pregnancy but before labour started?* | *YES* *1*  *NO* *2*  *DON’T KNOW* *8* | | | | | *6506*  *6506* |
| *6502* | *Did the bleeding stain her clothes, the bed or the floor?* | *CLOTHES*  *8 BED*  *8 FLOOR* | *YES 1* | *1* | *NO 2*  *1* | *DK*  *2* |  |
| *6504* | *Was she in pain while bleeding?* | *2* *8*  *YES* *1*  *NO* *2*  *DON’T KNOW* *8* | | | | |  |

| **NO.** | **QUESTIONS AND FILTERS** | | **CODING CATEGORIES** | **SKIP** |
| --- | --- | --- | --- | --- |
| 6506 | Did she have other episodes of bleeding during this pregnancy? | | YES 1  NO 2  DON’T KNOW 8 | 6508  6508 |
| 6507 | Were those episodes of bleeding painful? | | YES 1  NO 2  DON’T KNOW 8 |  |
| 6508 | Did she have a vaginal examination during her illness? | | YES 1  NO 2  DON’T KNOW 8 | 6701  6701 |
| 6509 | Did the vaginal examination increase the bleeding? | | YES 1  NO 2  NOT APPLICABLE (no bleeding) 7  DON’T KNOW 8 |  |
| 6701 | Was any attempt made during her pregnancy to induce abortion or to terminate the pregnancy? | | YES 1  NO 2  DON’T KNOW 8 |  |
| 6702 | Did the woman do MR? | | YES 1  NO 2  DON’T KNOW 8 |  |
| 6703 | CHECK Q6701 AND Q6702. | Q6701 CODED YES OR Q6702 CODED YES 1  Q6701 NOT CODED YES AND Q6702 NOT CODED YES 2 | | 6801 |
| 6704 | Whose help did she seek to induce abortion or to terminate the pregnancy or to do MR? | | HEALTH PROFESSIONAL  QUALIFIED DOCTOR (MBBS) A  NURSE/MIDWIFE/PARAMEDIC B  FAMILY WELFARE VISITOR C  COMMUNITY SKILLED BIRTH ATTENDANT D  MA/SACMO E  HEALTH ASSISTANT F  FAMILY WELFARE ASSISTANT G  OTHER PROVIDER  TRAINED TBA H  UNTRAINED TBA I  UNQUALIFIED DOCTOR J  TRADITIONAL HEALER (HERBALIST, HOMEOPATH, SPIRITUAL HEALER) K  RELATIVE/FRIENDS L  OTHER NGOs  BRAC SHASTHAY SEBIKA M  OTHER SHASTHA SEBIKA N  OTHER FIELD WORKER O  OTHER X  (SPECIFY)  DON’T KNOW/UNSURE Y  NONE - SELF INDUCED Z |  |
| 6705 | Was any foreign object inserted inside the woman to induce abortion or to terminate the pregnancy or to do MR? | | YES 1  NO 2  DON’T KNOW 8 | 6707  6707 |
| 6706 | What object was inserted? | | STICK A  TUBES B  SYRINGES C  OTHERS X  DON’T KNOW Y |  |

| ***NO.*** | ***QUESTIONS AND FILTERS*** | ***CODING CATEGORIES*** | ***SKIP*** |
| --- | --- | --- | --- |
| *6707* | *Did the woman take any drugs or injections, or eat anything to induce abortion or to terminate the pregnancy or to do MR?* | *YES* *1*  *NO* *2*  *DON’T KNOW* *8* | *6709*  *6709* |
| *6708* | *What drugs or injections did she take?*  *Verbatim*  *(1)*  *(2)* | *\|* *\|* *\| \|* *\|* *\|*  *DON'T KNOW/UNSURE* *98* |  |
| *6709* | *Did she have any of the following after inducing abortion or terminating her pregnancy or doing MR? [please read the choices and probe]*  *Foul-smelling discharge Fever*  *Abdominal distention*  *Severe bleeding* | *Don’t*  *Yes No Know Foul-smelling discharge ...... 1............ 2* *8*  *Fever ................................... 1............ 2* *8*  *Abdominal distention ........... 1............ 2* *8*  *Severe bleeding .................. 1............ 2* *8* |  |
| *6801* | *Did she have a pregnancy prior to the last one before death?* | *YES* *1*  *NO* *2* | *8901* |
|  | *THE FOLLOWING QUESTIONS (Q6802-Q6807) REFERS TO ALL PREVIOUS PREGNANCIES PRIOR TO THE LAST ONE BEFORE DEATH* |  |  |
| *6802* | *Did she ever have any complication in a previous pregnancy?* | *YES* *1*  *NO* *2*  *DON’T KNOW* *8* |  |
| *6803* | *Did she have a cesarean section in a previous pregnancy?* | *YES* *1*  *NO* *2*  *DON’T KNOW* *8* |  |
| *6804* | *Did she have Forcep / Ventos in a previous pregnancy? (Interviewer: explain to respondents what Forcep/Ventos means)* | *YES, FORCEP* *1*  *YES, VENTOS* *2*  *YES, BOTH FORCEP & VENTOS* *3*  *NO* *4*  *DON'T KNOW/UNSURE* *8* |  |
| *6805* | *Did*  *(NAME) ever have any still births in a previous pregnancy? If yes, how many?*  *(If none, write =0)* | *Times* *\|* *\|*  *DON’T KNOW* *8* |  |
| *6806* | *Did*  *(NAME) ever have any miscarriages/abortions in a previous pregnancy? If yes, how many times?*  *(If none, write =0)* | *Times* *\|* *\|*  *DON’T KNOW* *8* |  |
| *6807* | *Did*  *(NAME) ever have any MRs in a previous pregnancy? If yes, how many times? (If none, write =0)* | *Times* *\|* *\|*  *DON’T KNOW* *8* | *8901*  *8901* |
| *6807a* | *Did she die during an abortion?* | *YES* *1*  *NO* *2*  *DON’T KNOW* *8* |  |

**SECTION 7**

**MODULE 2: FOR DEATHS DURING LABOUR, DELIVERY OR AFTER DELIVERY**

| **NO.** | **QUESTIONS AND FILTERS** | **CODING CATEGORIES** | **SKIP** |
| --- | --- | --- | --- |
| 7101 | Did (NAME) ever see anyone for a medical checkup during the last pregnancy before she died? | YES 1  NO 2  DON’T KNOW 8 | 7201  7201 |
| 7102 | From whom did she receive the medical checkup when she was pregnant?  IF YES: Whom did she see?  Anyone else?  PROBE TO IDENTIFY EACH TYPE OF PERSON AND RECORD ALL MENTIONED.  IF CODE ‘D’ CIRCLED (WRITE NAME OF CSBA) | HEALTH PERSONNEL |  |
|  |  | QUAL. DOCTOR… A |  |
|  |  | NURSE/MIDWIFE/PARAMEDIC… B |  |
|  |  | FAMILY WELFARE VISITOR… C |  |
|  |  | COMMUNITY SKILLED |  |
|  |  | BIRTH ATTENDANT… D |  |
|  |  | MA/SACMO… E |  |
|  |  | COMMUNITY HEALTH |  |
|  |  | CARE PROVIDER… F |  |
|  |  | HEALTH ASST… G |  |
|  |  | FAMILY WELFARE |  |
|  |  | ASSISTANT… H |  |
|  |  | NGO WORKER. I |  |
|  |  | OTHER PERSON |  |
|  |  | TRAINED TBA… J |  |
|  |  | UNTRAINED TBA… K |  |
|  |  | UNQUALIFIED DOCTOR… L |  |
|  |  | OTHER X |  |
|  |  | (SPECIFY) |  |
| 7103 | Did she first seek medical checkup during her last pregnancy because | BECAUSE OF PROBLEM ONLY 1 |  |
|  | she had a problem or just for a checkup? | FOR CHECK UP ONLY 2 | 7105 |
|  |  | FOR BOTH 3 |  |
|  |  | DON’T KNOW 8 | 7105 |
| 7104 | For what problem did she first seek medical checkup during her last pregnancy?  Verbatim | \| \| \| \| \| \|  DON'T KNOW/UNSURE 98 |  |
|  | (1) |  |  |
|  | (2)  _ |  |  |
| 7105 | How many months pregnant was she at the time of her first medical checkup during her last pregnancy? | MONTHS \| \| \| DON'T KNOW/UNSURE 98 |  |
| 7106 | How many times did she get medical check-up during her last pregnancy? | NUMBER OF TIMES \| \| \| DON'T KNOW/UNSURE 98 |  |
| 7201 | Did she have swelling around her ankles during her pregnancy? | YES 1  NO 2  DON’T KNOW 8 |  |
| 7202 | Did she have puffiness of the face during her pregnancy? | YES 1  NO 2  DON'T KNOW/UNSURE 8 |  |
| 7203 | Did she complain of blurred vision during her pregnancy? | YES 1 |  |
|  |  | NO 2 | 7204 |
|  |  | DON’T KNOW 8 | 7204 |
| 7203a | During the last 3 months of pregnancy did she suffer from blurred vision? | YES 1  NO 2  DON’T KNOW 8 |  |
| 7204 | Did she have her blood pressure measured during her pregnancy? | YES 1 |  |
|  |  | NO 2 | 7206 |
|  |  | DON’T KNOW 8 | 7206 |

| ***NO.*** | ***QUESTIONS AND FILTERS*** | ***CODING CATEGORIES*** | ***SKIP*** |
| --- | --- | --- | --- |
| *7205* | *Do you know whether her blood pressure was normal or high or low?* | *NORMAL* *1*  *HIGH* *2*  *LOW* *3*  *DON’T KNOW* *8* |  |
| *7206* | *Did she have any loss of consciousness during her last illness?* | *YES* *1* |  |
|  |  | *NO* *2* | *7207* |
|  |  | *DON’T KNOW* *8* | *7207* |
| *7206a* | *For how many days did she have loss of consciousness?* | *\|* *\|* *\|*  *days*  *DON'T KNOW/UNSURE* *9998* |  |
| *7206b* | *Did the unconsciousness continue until death?* | *YES* *1*  *NO* *2*  *DON’T KNOW* *8* |  |
| *7207* | *Did she have fits (convulsions) during her last illness?* | *YES* *1* |  |
|  |  | *NO* *2* | *7209* |
|  |  | *DON’T KNOW* *8* | *7209* |
| *7208* | *How many days/months before her death did the fits start?*  *(Write in months and days. If less than 1 month, then write 00 for months and only write in days)* | *START \|* *\|* *\| \|* *\|* *\|*  *months days*  *DON'T KNOW/UNSURE* *9998* |  |
| *7209* | *Did she have headache during her last illness?* | *YES* *1* |  |
|  |  | *NO* *2* | *7301* |
|  |  | *DON'T KNOW/UNSURE* *8* | *7301* |
| *7210* | *Was the headache continuous or on and off?* | *CONTINUOUS* *1*  *ON AND OFF* *2*  *DON'T KNOW/UNSURE* *8* |  |
| *7211* | *How was the headache? Severe, moderate, mild, or sometimes mild and sometimes severe?* | *SEVERE* *1*  *MODERATE* *2* |  |
|  |  | *MILD* *3* |  |
|  |  | *SOMETIMES MILD AND SOMETIMES SEVERE* *4* |  |
|  |  | *DON'T KNOW/UNSURE* *8* |  |
| *7301* | *Did*  *(NAME) have fever during her last illness?* | *YES* *1* |  |
|  |  | *NO* *2* | *7306* |
|  |  | *DON’T KNOW* *8* | *7306* |
| *7302* | *How many days/months before her death did the fever start and end?*  *(Write in months and days. If less than 1 month, then write 00 for months and only write in days)* | *START \|* *\|* *\| \|* *\|* *\|*  *months days*  *END \|* *\|* *\| \|* *\|* *\|*  *months days* |  |
|  |  | *DIED WITH FEVER.* *9995* |  |
|  |  | *DON'T KNOW/UNSURE* *9998* |  |
| *7303* | *How was the fever like high or mild?* | *HIGH* *1*  *MILD* *2*  *DON'T KNOW/UNSURE* *8* |  |
| *7304* | *Was the fever continuous or on and off?* | *CONTINUOUS* *1*  *AFTER EVERY 1 - 2 DAYS* *2*  *AT NIGHT ONLY* *3*  *OTHER(specify)*  *.* *7*  *DON'T KNOW/UNSURE* *8* |  |

| **NO.** | **QUESTIONS AND FILTERS** | **CODING CATEGORIES** | **SKIP** |
| --- | --- | --- | --- |
| 7305 | Did the fever come with severe chills? | YES 1  NO 2  DON'T KNOW/UNSURE 8 |  |
| 7306 | Did the colour of her eye change to yellow (jaundice) during her last illness? | YES 1  NO 2  DON'T KNOW/UNSURE 8 |  |
| 7307 | Did she have itching of skin at any time during her last illness? | YES 1  NO 2  DON'T KNOW/UNSURE 8 |  |
| 7308 | Did her eyes, face or palms look pale (anaemic) during her last illness? | **YES NO DK**  PALE EYES 1 2 8  PALE FACE 1 2 8  PALE PALM 1 2 8 |  |
| 7309 | Did she have a cough during her last illness? | YES 1 |  |
|  |  | NO 2 | 7313 |
|  |  | DON’T KNOW 8 | 7313 |
| 7310 | How many days or months before her death did the cough start?  *(Write in months and days. If less than 1 month, then write 00 for months and only write in days)* | START \| \| \| \| \| \|  mons days  DON'T KNOW/UNSURE 9998 |  |
| 7311 | Did the cough produce sputum? | YES 1  NO 2  DON'T KNOW/UNSURE 8 |  |
| 7312 | Did she cough blood? | YES 1  NO 2  DON'T KNOW/UNSURE 8 |  |
| 7313 | Did she have difficulty in breathing during her last illness? | YES 1 |  |
|  |  | NO 2 | 7319 |
|  |  | DON’T KNOW 8 | 7319 |
| 7314 | Was the difficulty in breathing continuous or on and off? | CONTINUOUS 1  ON AND OFF 2  DON'T KNOW/UNSURE 8 |  |
| 7315 | How many days/months before her death did the difficulty in breathing start and end? | START \| \| \| \| \| \|  months days |  |
|  | *(Write in months and days. If less than 1 month, then write 00 for months and only write in days)* | END \| \| \| \| \| \|  months days |  |
|  |  | DID NOT IMPROVE/ Continued till death . 9995 |  |
|  |  | DON'T KNOW/UNSURE 9998 |  |
| 7316 | Was she breathless even on light work?  *(Except what is normally seen in late pregnancy, if applicable)* | YES 1  NO 2  DON'T KNOW/UNSURE 8 |  |
| 7317 | Was she breathless on lying on her back?  *(Except what is normally seen in late pregnancy, if applicable)* | YES 1  NO 2  DON'T KNOW/UNSURE 8 |  |
| 7318 | Was there pain in the chest with breathing? | YES 1  NO 2  DON’T KNOW 8 |  |
| 7319 | Did she have palpitations during her last illness? | YES 1  NO 2  DON'T KNOW/UNSURE 8 |  |

| **NO.** | **QUESTIONS AND FILTERS** | **CODING CATEGORIES** | **SKIP** |
| --- | --- | --- | --- |
| 7320 | Did she have chest pain during her last illness? | YES 1 |  |
|  |  | NO 2 | 7326 |
|  |  | DON’T KNOW 8 | 7326 |
| 7321 | Was the pain mild, moderate or severe? | SEVERE 1  MODERATE 2  MILD 3  DON'T KNOW/UNSURE 8 |  |
| 7322 | Did the pain start suddenly or gradually? | SUDDENLY 1  GRADUALLY 2  DON'T KNOW/UNSURE 8 |  |
| 7323 | Was the pain continuous or on and off? | CONTINUOUS 1  ON AND OFF 2  DON'T KNOW/UNSURE 8 |  |
| 7324 | How many days/months before her death did the pain start and end?  *(Write in months and days. If less than 1 month, then write 00 for months and only write in days)* | START \| \| \| \| \| \|  months days  END \| \| \| \| \| \|  months days |  |
|  |  | DID NOT IMPROVECONTINUED UNTIL DEATH 9995 |  |
|  |  | DON'T KNOW/UNSURE 9998 |  |
| 7325 | When she had the chest pain, did she also have pain elsewhere in her body? If, yes, where else did she have pain at the same time? | SHOULDER A  NECK B  ARMS C  NO PAIN ANYWHERE D  OTHER(specify) X |  |
| 7326 | Did she have abdominal pain before her death? | YES 1 |  |
|  |  | NO 2 | 7329 |
|  |  | DON’T KNOW 8 | 7329 |
| 7327 | How many days/months before her death did the abdominal pain start and end? | START \| \| \| \| \| \| months days  END \| \| \| \| \| \| months days  DID NOT IMPROVE/CONTINUED UNTIL DEATH  . 9995  DON'T KNOW/UNSURE 9998 |  |
|  | *(Write in months and days. If less than 1 month, then write 00 for months and only write in days)* |  |  |
| 7328 | Was the pain mild, moderate or severe? | SEVERE 1  MODERATE 2  MILD 3  SOMETHIMES MILD/SOMETIMES MORE 4  DON'T KNOW/UNSURE 8 |  |
| 7329 | Was there any change in the color of her urine before death? | YES 1 |  |
|  |  | NO 2 | 7331 |
|  |  | DON’T KNOW 8 | 7331 |
| 7330 | What color did the urine become? | LIGHT YELLOW 1  DARK YELLOW 2  CHUNER PANI (CLOUDY) 3  BHATER MAAR (THICK-WHITE) 4  BLOOD STAINED/RED 5  OTHER(specify)  . 7  DON'T KNOW/UNSURE 8 |  |

| **NO.** | **QUESTIONS AND FILTERS** | **CODING CATEGORIES** | | | | | **SKIP** |
| --- | --- | --- | --- | --- | --- | --- | --- |
| 7331 | Was there any change in her daily frequency of urine before her death? | YES 1  NO 2  DON’T KNOW 8 | | | | | 7401  7401 |
| 7332 | Compared to before, how many times was she passing urine in a day - more than before, less than before, or no urine at all? | MORE THAN BEFORE 1  LESS THAN BEFORE 2  NO URINE AT ALL 3  DON'T KNOW/UNSURE 8 | | | | |  |
| 7333 | Since how many days/months before her death did she start to pass urine (ANSWER TO Q7332 )?  *(Write in months and days. If less than 1 month, then write 00 for months and only write in days)* | START \| \| \| \| \| \|  months days  DON'T KNOW/UNSURE 9998 | | | | |  |
| 7401 | During her last illness, did she have leaking membrane? | YES 1  NO 2  DON’T KNOW 8 | | | | | 7404  7404 |
| 7402 | How many days/months before her death did she have leaking membrane?  *(Write in months and days. If less than 1 month, then write 00 for months and only write in days)* | START \| \| \| \| \| \|  months days  DON'T KNOW/UNSURE 9998 | | | | |  |
| 7403 | Was she in pain when she had leaking membrane? | YES 1  NO 2  DON’T KNOW 8 | | | | |  |
| 7404 | Did she have other episodes of leaking membrane during her last pregnancy? | YES 1  NO 2  DON’T KNOW 8 | | | | | 7501  7501 |
| 7405 | Were these episodes of leaking membrane during her last pregnancy painful? | YES 1  NO 2  DON’T KNOW 8 | | | | |  |
| 7501 | Did she have bleeding from the vagina during her last pregnancy? | YES 1  NO 2  DON’T KNOW 8 | | | | |  |
| 7501a | Was there vaginal bleeding during the first 6 months of pregnancy? | YES 1  NO 2  DON’T KNOW 8 | | | | |  |
| 7501b | Was there vaginal bleeding during the last 3 months of pregnancy but before labour started? | YES 1  NO 2  DON’T KNOW 8 | | | | | 7506  7506 |
| 7502 | Did the bleeding stain her clothes, the bed or the floor? | CLOTHES  8 BED  8 FLOOR | YES 1 | 1 | NO 2  1 | DK  2 |  |
| 7503 | Did the bleeding start before the birth of the child? | 2 8  YES 1  NO 2  DON’T KNOW 8 | | | | |  |
| 7504 | Was she in pain while bleeding (not menses)? | YES 1  NO 2  DON’T KNOW 8 | | | | |  |
| 7505 | Did the pain start before the labour pains started? | YES 1  NO 2  DON’T KNOW 8 | | | | |  |
| 7506 | Did she have other episodes of bleeding during this pregnancy? | YES 1  NO 2  DON’T KNOW 8 | | | | | 7508  7508 |

| **NO.** | **QUESTIONS AND FILTERS** | | **CODING CATEGORIES** | **SKIP** |
| --- | --- | --- | --- | --- |
| 7507 | Were those episodes of bleeding painful? | | YES 1  NO 2  DON’T KNOW 8 |  |
| 7508 | Did she have a vaginal examination during her last pregnancy? | | YES 1 |  |
|  |  | | NO 2 | 7601 |
|  |  | | DON’T KNOW 8 | 7601 |
| 7509 | Did the vaginal examination increase the bleeding? | | YES 1  NO 2  DON’T KNOW 8 |  |
| 7601 | How many hours or days before her death did her labour pain start?  *(If less than 1 day, then write in hours, if 1 or more days then write in completed days)* | | HOURS ....................... 1 \| \| \|  DAYS........................... 2 \| \| \|  DON'T KNOW/UNSURE 998 |  |
| 7603 | Where did she give birth? | | HOME |  |
|  |  | | HOME 11 |  |
|  | PROBE TO IDENTIFY THE TYPE OF SOURCE AND CIRCLE THE APPROPRIATE CODE. | | PUBLIC SECTOR  PUBLIC HOSPITAL… 21 |  |
|  |  | | DIST. HOSP 22 |  |
|  | IF UNABLE TO DETERMINE IF A HOSPITAL, HEALTH CENTER, OR  CLINIC IS PUBLIC OR PRIVATE MEDICAL, WRITE THE NAME OF THE PLACE. | | MCWC… 23  UPAZILLA HEALTH COMPLEX 24 |  |
|  |  | | UH & FAMILY WELFARE CENTRE……25 |  |
|  |  | | COM. CLINIC 28 |  |
|  | (NAME OF THE PLACE) | | OTHER PUBLIC |  |
|  |  | | SECTOR (SPECIFY) 26 |  |
|  |  | | NGO SECTOR |  |
|  |  | | NGO STATIC CLINIC… 31 |  |
|  |  | | DELIVERY HUT 36 |  |
|  |  | | PRIVATE MED. SECTOR |  |
|  |  | | PVT. HOSPITAL/CLINIC… 41 |  |
|  |  | | OTHER (SPECIFY) 96 |  |
| 7604 | Who conducted the delivery? | | HEALTH PERSONNEL  QUAL. DOCTOR A  NURSE/MIDWIFE/PARAMEDIC B  FAMILY WELFARE VISITOR… C  COMMUNITY SKILLED  BIRTH ATTENDANT D  MA/SACMO E  COMMUNITY HEALTH  CARE PROVIDER… F  HEALTH ASST G  FAMILY WELFARE ASSISTANT H  NGO WORKER I  OTHER PERSON:  TRAINED TBA J  UNTRAINED TBA K  UNQUALIFIED DOCTOR… L  RELATIVES M  NEIGHBORS/FRIENDS N  OTHER X (SPECIFY)  NO ONE ASSISTED… Y |  |
|  | Anyone else? | |  |  |
|  | PROBE FOR THE TYPES OF PERSON(S) AND RECORDALL MENTIONED. | |  |  |
|  | IF RESPONDENT SAYS NO ONE ASSISTED, PROBE TO DETERMINE WHETHER ANY ADULTS WERE PRESENT AT THE DELIVERY. | |  |  |
|  | IF CODE ‘D’ CIRCLED: | |  |  |
|  | (WRITE NAME OF CSBA) | |  |  |
|  | (WRITE NAME OF CSBA) | |  |  |
| 7605 | During the delivery, were/was *(topic)*:   1. Instruments used to help the baby out (forceps/ventose) 2. An operation done to get the baby out (cesarean section) 3. A blood transfusion given 4. A saline infusion given 5. Hysterectomy for rupture uterus 6. Manual removal of placenta | **YES NO DK**  FORCEPS/VACUUM ------------------- 1 --------- 2 8  CESAREAN SECTION----------------- 1 --------- 2 8  BLOOD TRANSFUSION--------------- 1 --------- 2 8  SALINE INFUSION---------------------- 1 --------- 2 8  HYSTERECTOMY ---------------------- 1 --------- 2 8  MANUAL PLACENTA REMOVAL--- 1 --------- 2 8 | |  |

| **NO.** | **QUESTIONS AND FILTERS** | **CODING CATEGORIES** | **SKIP** |
| --- | --- | --- | --- |
| 7606 | How long was she in labour for?  *(if less than 1 hour write 00)* | \| \| \| HOURS NEVER IN LABOUR (C-SECTION) 95  DON’T KNOW 98 | 7610  7608 |
| 7607 | Do you think she had prolonged labour? | YES 1  NO 2  DON'T KNOW/UNSURE 8 |  |
| 7608 | Did she have excessive bleeding during labour or delivery? | YES 1  NO 2  DON'T KNOW/UNSURE 8 | 7610  7610 |
| 7609 | Did the bleeding stain her clothes, the bed or the floor? | YES NO DK  CLOTHES 1 2 8  BED 1 2 8  FLOOR 1 2 8 |  |
| 7610 | Were any drugs used just before or during the labour? | YES 1 |  |
|  |  | NO 2 | 7613 |
|  |  | NOT APPLICABLE (no labour pain) 7 | 7613 |
|  |  | DON'T KNOW/UNSURE 8 | 7613 |
| 7611 | Can tell me the name of the drugs that was used? | DRUG.................................... \| \| \| \|  DRUG.................................... \| \| \| \|  DRUG.................................... \| \| \| \|  DRUG.................................... \| \| \| \|  DRUG.................................... \| \| \| \| DONOTT KNOW/UNSURE 998 |  |
| 7612 | What were the routes/modes used to give the drugs? | ORAL A  INTRAMUSCULAR B  INTRAVENOUS C  OTHER (specify ) X DON'T KNOW/UNSURE Y |  |
| 7613 | How many days or months before her death did she deliver? | HOURS ....................... 1 \| \| \| |  |
|  | *(If less than 1 day then write in hours, if less than 30 days write in days and if more, then in completed months)* | DAYS........................... 2 \| \| \| |  |
|  |  | MONTHS..................... 3 \| \| \| |  |
|  |  | NEVER DELIVERED. 997 | 7626 |
|  |  | DON'T KNOW/UNSURE 998 |  |
| 7614 | Did she have difficulty in delivering the baby? | YES 1  NO 2  DON'T KNOW/UNSURE 8 |  |
| 7615 | What part of the baby came out first? | HEAD 1  LEGS 2  SHOULDER 3  ARMS 4  FACE 5  CESAREAN SECTION 6  DON'T KNOW/NOT SURE 8 | 7621 |
| 7616 | Did she have difficulty in delivering the placenta? | YES 1  NO 2  DIED BEFORE PLACENTA WAS  DELIVERED… 3  DON'T KNOW/UNSURE 8 | 7620 |

| **NO.** | **QUESTIONS AND FILTERS** | **CODING CATEGORIES** | **SKIP** |
| --- | --- | --- | --- |
| 7617 | How long after the birth of the child was the placenta delivered?  *(If less than 1 hour write 00)* | \| \| \| HOURS  DON'T KNOW/UNSURE 98 |  |
| 7618 | Was manual removal of the placenta done? | YES 1  NO 2  DON'T KNOW/UNSURE 8 |  |
| 7619 | Was the placenta delivered completely or partially? | COMPLETELY 1  PARTIALLY 2  DON’T KNOW 8 |  |
| 7620 | Did she need to be hospitalized to deliver the placenta? | YES 1  NO 2  HAD ALREADY BEEN ADMITTED  IN HOSPITAL… 3  DON'T KNOW/UNSURE 8 |  |
| 7621 | Did she have too much bleeding after the baby was born? | YES 1  NO 2  DON'T KNOW/UNSURE 8 | 7623  7623 |
| 7622 | Did the bleeding stain her clothes, the bed or the floor? | YES NO DK  CLOTHES 1 2  8 BED 1 2  8 FLOOR 1 |  |
| 7623 | Did she have foul-smelling discharge from the vagina after the baby was born? | 2 8  YES 1  NO 2  DON’T KNOW 8 |  |
| 7623a | Did she have foul-smelling discharge from the vagina during delivery? | YES 1  NO 2  DON’T KNOW 8 |  |
| 7624 | Did she have pain in the legs after the baby was born? | YES 1  NO 2  DON’T KNOW 8 |  |
| 7625 | Did she have fever after the baby was born? | YES 1  NO 2  DON’T KNOW 8 |  |
| 7626 | Did she have fits (convulsions) during her pregnancy or before delivery of the baby during labor? | YES 1  NO 2  DON’T KNOW 8 | 7628  7628 |
| 7627 | Did the fits stop after the baby was born? | YES 1  NO 2  NEVER DELIVERED. 3  DON’T KNOW 8 | 7628a  7801  7628a |
| 7628 | Did she develop fits (convulsions) after the baby was born? | YES 1  NO 2  DON’T KNOW 8 |  |
| 7628a | Did she die during or after a multiple pregnancy? | YES 1  NO 2  DON’T KNOW 8 |  |
| 7628b | Was she breastfeeding the child in the days before death? | YES 1  NO 2  DON’T KNOW 8 |  |

| **NO.** | **QUESTIONS AND FILTERS** | **CODING CATEGORIES** | **SKIP** |
| --- | --- | --- | --- |
| 7629 | Did the colour of her eyes become yellow(jaundice) after delivery? | YES 1  NO 2  DON'T KNOW/UNSURE 8 | 7801  7801 |
| 7630 | How many days after delivery did her eyes become yellow(jaundice)? | \| \| \| \| DAYS DON'T KNOW/UNSURE 998 |  |
| 7801 | Did she have a pregnancy prior to the last one before death? | YES 1  NO 2 | 8901 |
|  | THE FOLLOWING QUESTIONS (Q7802-Q7807) REFERS TO ALL PREVIOUS PREGNANCIES PRIOR TO THE LAST ONE BEFORE DEATH |  |  |
| 7802 | Did she ever have any complication in a previous pregnancy? | YES 1  NO 2  DON’T KNOW 8 |  |
| 7803 | Did she have a cesarean section in a previous pregnancy? | YES 1  NO 2  DON’T KNOW 8 |  |
| 7804 | Did she have Forcep/ Ventos in a previous pregnancy? (Interviewer: explain to respondents what Forcep/Ventos means) | YES, FORCEP 1  YES, VENTOS 2  YES, BOTH FORCEP & VENTOS 3  NO 4  DON'T KNOW/UNSURE 8 |  |
| 7805 | Did (NAME) ever have any still births in a previous pregnancy? If yes, how many?  (If none, write =0) | Times \| \|  DON’T KNOW 8 |  |
| 7806 | Did (NAME) ever have any miscarriages/abortions in a previous pregnancy? If yes, how many times?  (If none, write =0) | Times \| \|  DON’T KNOW 8 |  |
| 7807 | Did (NAME) ever have any MR in a previous pregnancy? If yes, how many times?  (If none, write =0) | Times \| \|  DON’T KNOW 8 | 8901  8901 |
